# Six novel nutritional-related indicators predict 3-year all-cause mortality among community-dwelling older adults in China: A cohort study based on CLHLS from 2014 to 2018

**DOI:** 10.1097/MD.0000000000048952

**Published:** 2026-05-22

**Authors:** Tian Hu, Yuhan Ye, Zelong Zhao, Zheng Hu

**Affiliations:** a Department of Rehabilitation Medicine, JieYang People’s Hospital, Jieyang, Guangdong, China; b Child Developmental and Behavior Center, The Third Affiliated Hospital of Sun Yat-sen University, Guangzhou, Guangdong, China; c Department of Orthopedic Trauma, JieYang People’s Hospital, Jieyang, Guangdong, China.

**Keywords:** all-cause mortality, Chinese Longitudinal Healthy Longevity Survey, cohort study, geriatric nutritional risk index, nutrition-related indicators

## Abstract

Malnutrition is a modifiable risk factor for mortality among older adults. However, simple and objective nutritional assessment tools remain limited in community settings. This study examined the associations between 6 nutrition-related indicators and all-cause mortality, aiming to identify the most predictive indicator. Data were obtained from the 2014 to 2018 wave of the Chinese Longitudinal Healthy Longevity Survey. Six nutrition-related indicators were examined: hemoglobin-albumin-lymphocyte-platelet (HALP) score, prognostic nutritional index (PNI), cholesterol-modified PNI (CPNI), triglyceride-total cholesterol-body weight index (TCBI), geriatric nutritional risk index (GNRI), and blood urea nitrogen to serum albumin ratio. Participants were categorized into tertiles for each indicator. Cox proportional hazards models, Kaplan–Meier curves, time-dependent receiver operating characteristic curves, and restricted cubic spline analyses were applied. A total of 1691 older adults were included, and 578 deaths occurred over a median follow-up of 3.3 years. Compared with the lowest tertile, the highest tertile of HALP score, PNI, TCBI, and GNRI were associated with lower mortality risk. In contrast, the highest tertile of CPNI and blood urea nitrogen to serum albumin ratio predicted higher mortality risk. Among these indicators, GNRI demonstrated the highest discriminative performance. restricted cubic spline analyses revealed significant nonlinear dose-response relationships between all-cause mortality and HALP score, CPNI, and TCBI. In conclusion, all 6 indicators were significantly associated with mortality risk, with the GNRI providing more effective predictive value among community-dwelling older adults.

## 1. Introduction

China is experiencing rapid population aging, with individuals aged 65 and older accounting for 13.5% of the population in 2020, a proportion projected to rise significantly in the coming decades.^[1]^ This demographic shift poses major public health challenges, especially for managing age-related diseases and improving quality of life. Chinese seniors also face distinct challenges such as unequal healthcare access, socioeconomic vulnerabilities, and regional inequities in health resources.^[2–4]^ Consequently, there is a pressing need to identify modifiable risk factors to alleviate adverse health outcomes in this growing population.

Malnutrition is a widespread yet modifiable concern, affecting approximately 25% of community-dwelling older adults in China.^[5]^ It is collectively driven by age-related declines in appetite, digestion and nutrient absorption.^[6,7]^ Mounting evidence links malnutrition to adverse outcomes, including frailty, delayed recovery, and increased mortality.^[8–10]^ However, despite its prognostic significance, systematic screening for nutritional risk remains underprioritized in the current diagnostic and therapeutic workup.^[11]^ While instruments like the Mini Nutritional Assessment show research utility,^[12]^ operational complexities involving multi-domain scoring and subjective patient-reported parameters limit their feasibility for large population implementation.^[13]^ These findings underscore the pressing need for a valid, straightforward nutritional risk screening tool tailored for community-dwelling older adults in China.

Laboratory biomarkers are gaining popularity as prognostic tools in clinical settings, frequently superseding complex multivariate scoring systems, thanks to their cost-effectiveness and wide accessibility. Recent advances have introduced various innovative nutrition-related indicators, such as hemoglobin-albumin-lymphocyte-platelet (HALP) score, prognostic nutritional index (PNI), cholesterol-modified PNI (CPNI), triglyceride-total cholesterol-body weight index (TCBI), geriatric nutritional risk index (GNRI), and blood urea nitrogen to serum albumin ratio (BAR). These composite indicators, derived from routine hematological and biochemical parameters, provide a more precise and holistic assessment of an individual’s nutritional status compared to single biomarkers. Emerging clinical evidence indicates that HALP score, PNI, CPNI, TCBI, GNRI, and BAR are widely employed in nutritional evaluation of various geriatric conditions, such as cancer, stroke, and heart failure.^[14–19]^ However, despite previous investigations,^[20–22]^ the reliability of their association with age-related all-cause mortality remains uncertain, owing to their nonspecific nature and variability across different populations. Therefore, further validation of these nutritional indicators is warranted, particularly within community-dwelling older adults in China.

In this study, we utilized data from the 2014 to 2018 waves of the Chinese Longitudinal Healthy Longevity Survey (CLHLS) to investigate the associations of HALP score, PNI, CPNI, TCBI, GNRI, and BAR with all-cause mortality among community-dwelling older adults in China. Furthermore, we sought to identify the optimal nutritional predictor of mortality within this population.

## 2. Materials and method

### 2.1. Data source

This analysis utilized longitudinal data from the CLHLS, a nationally representative prospective cohort established by Peking University in 1998 to examine the synergistic effects of social, behavioral, environmental, and biological determinants on aging trajectories. The CLHLS conducts triennial to quadrennial follow-ups across 23 provincial-level regions in China and has cumulatively surveyed more than 113,000 households. Sampling design, data quality assessments, and other methodological details of the CLHLS have been documented in prior publications.^[23]^

### 2.2. Study population

**Figure 1.**
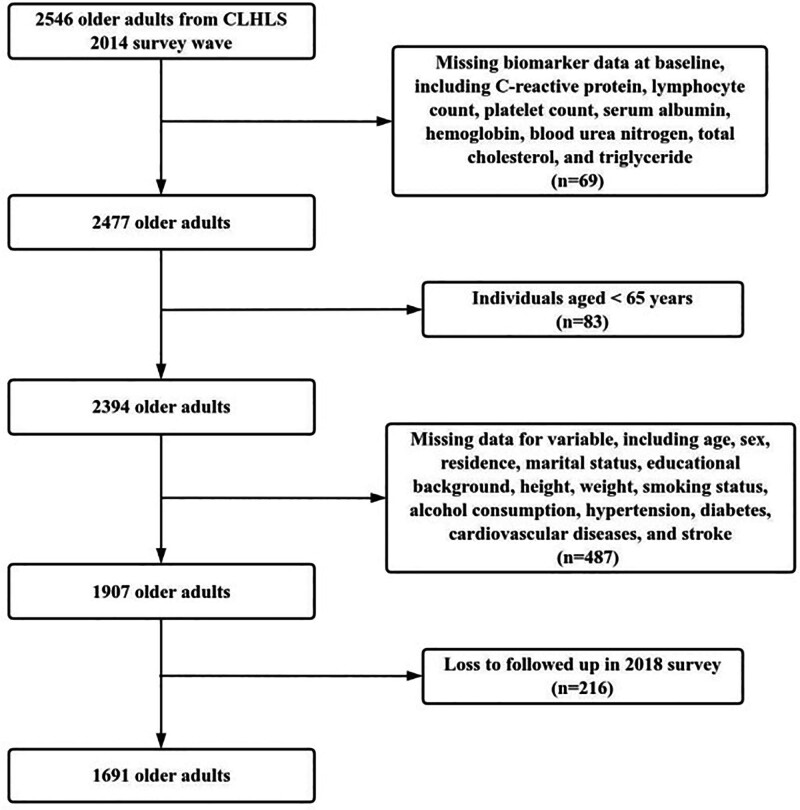
Flowchart of inclusion of older participants. CLHLS = Chinese Longitudinal Healthy Longevity Survey, n = number of participants.

This study analyzed data from the 2014 to 2018 waves. Of the 2546 participants enrolled in 2014, 1691 were included in the final analysis after applying the following exclusion criteria: missing baseline biomarker data (n = 69); age < 65 years at enrollment (n = 83); incomplete covariate information (n = 487); and loss to follow-up (n = 216). The enrollment details were summarized in Figure 1. Ethical approval was obtained from the Research Ethics Committee of Peking University (IRB00001052-13074). All senior participants or their guardians were informed about the survey content and provided informed consent.

### 2.3. Measurement

#### 2.3.1. Assessment of exposure variable

At baseline, 5 mL of fasting venous blood was collected from older participants using heparin anticoagulant vacuum tubes by trained personnel. Samples were centrifuged onsite, stored at −20°C, and transported to the Clinical Laboratory Center of Capital Medical University for routine hematological and biochemical testing. Key biomarkers included serum albumin, hemoglobin, C-reactive protein, blood urea nitrogen, total cholesterol, and triglycerides, using standardized automated methods.

According to previous studies, nutritional indicators were calculated from the lymphocyte count, platelet count, serum albumin, hemoglobin, blood urea nitrogen, total cholesterol, and triglyceride using standardized equations. Detailed computational protocols for each nutrition-related indicator were provided in Table 1.

**Table 1:** Details of the nutrition-related indicators utilized in the study.

Exposure variable	Calculation formula	Reference
HALP score	Hemoglobin (g/L)×Albumin (g/L)× Lymphocyte count (109/L)÷Platelet count (109/L)	[14]
PNI	Albumin (g/L)+5 × Lymphocyte count (109/L)	[15]
CPNI	4.8×[Total cholesterol (mg/dL)÷38.67]− 1.5×Albumin (g/L)−7.7×Lymphocyte count (109/L)+126	[16]
TCBI	Triglyceride (mg/dL)×Total cholesterol (mg/dL) ×weight (kg)÷1000	[17]
GNRI	[1.489×Albumin (g/L)]+41.7×(present body weight÷ideal body weight) If present body weight exceed the ideal body weight, the value of “present body weight ÷ ideal body weight” set to 1.0 ideal body weight (male)=height(cm)-100-(height (cm)-150)÷4 ideal body weight (female)=height(cm)-100-(height (cm)-150)÷2.5	[18]
BAR	Blood urea nitrogen (mg/dL)÷[ Albumin (g/L)×0.1]	[19]

BAR = blood urea nitrogen to serum albumin ratio, CPNI = cholesterol-modified prognostic nutritional index, GNRI = geriatric nutritional risk index, HALP = hemoglobin-albumin-lymphocyte-platelet, PNI = prognostic nutritional index, TCBI = triglyceride-total cholesterol-body weight index.

#### 2.3.2. Assessment of mortality

During the 2018 follow-up, survival status was determined for all enrolled older adults. For deceased participants, the date of death and relevant details were collected from family members via questionnaires and interviews. Survival duration was calculated as the time (in months) from the baseline assessment to the date of death. Participants or their families who could not be contacted during the 2018 follow-up phase were classified as lost to follow-up. For surviving individuals, survival time was measured from the baseline assessment to the 2018 follow-up contact.

#### 2.3.3. Assessment of covariates

Baseline covariates were collected through a structured questionnaire, including age, sex, residence, marital status, educational background, body mass index (BMI), smoking status, alcohol consumption, hypertension, diabetes, cardiovascular diseases, and stroke. Age was stratified into ≤ 80 years and > 80 years. Marital status was categorized as married or single (unmarried/divorced/widowed). Educational background was categorized as illiteracy (no formal education) or literacy (≥ 1 year of schooling). BMI classifications followed Worls Hea;th Organization standards: underweight (< 18.5 kg/m^2^), normal (18.5–23.9 kg/m^2^), and overweight (≥ 24 kg/m^2^). Smoking status and alcohol consumption were defined as current, former, or never. Hypertension was diagnosed as systolic/diastolic blood pressure ≥ 140/90 mm Hg or self-reported clinician-confirmed hypertension. Diabetes was defined as fasting blood glucose ≥ 7.0 mmol/L or self-reported clinical diagnosis. Self-reported medical records were used to ascertain histories of cardiovascular diseases and stroke. Inflammatory status at baseline was assessed using C-reactive protein levels.

### 2.4. Statistical analysis

Continuous variables were presented as median with interquartile ranges, and categorical variables were expressed as counts (percentages). Baseline characteristics between survivor and non-survivor groups were compared using the Mann–Whitney *U* test for continuous variables and the *χ*^2^ test for categorical variables.

Kaplan–Meier curves with log-rank tests were used to compare cumulative survival probabilities across tertiles (T1-T3) of the 6 nutrition-related indicators. Cox proportional hazards model was used to investigate the association between each indicator and all-cause mortality, reporting hazard ratio (HR) with corresponding 95% confidence interval (CI) for 3 models: the crude model (unadjusted); adjusted model 1 (adjusted for age, sex, residence, marital status, educational background, BMI, smoking status and alcohol consumption); and adjusted model 2 (further adjusted for hypertension, diabetes, cardiovascular diseases, stroke and C-reactive protein levels based on adjusted model 1).

Nonlinear relationships between 6 nutrition-related indicators and all-cause mortality were modeled using restricted cubic splines with 4 knots at the 25th, 50th, 75th, and 95th percentiles. A nonlinear relationship was evaluated through likelihood ratio test. For indicators showing significant nonlinearity, inflection points were identified using 2-piecewise linear regression. Predictive performance of each indicators for all-cause mortality was evaluated using time-dependent receiver operating characteristic curves, with discrimination quantified by the area under the curve (AUC). Differences in AUC values between indicators were compared using DeLong test.

Older participants were stratified into low- and high-level groups based on the median values of each indicator. Subgroup analyses were performed to assess potential heterogeneity in associations across strata defined by age, sex, residence, BMI, hypertension, diabetes, cardiovascular diseases and stroke. Interaction *P* values were derived from likelihood ratio tests comparing regression models with and without interaction terms. Sensitivity analyses were performed to verify the robustness of the findings, including: exclusion of participants aged over 100 years, and removal of individuals who died within 1 year of follow-up.

All statistical analyses were conducted using R software (version 4.1.3; https://cran.r-project.org/), R Studio (version 2024.12.1+563; https://posit.co/products/open-source/rstudio), and IBM SPSS (version 26.0; IBM Corp., Armonk). A 2-sided *P* < .05 was considered statistically significant.

## 3. Results

### 3.1. Baseline characteristics

A total of 1691 older adults were included, with 578 deaths (34.2%) recorded over a median follow-up of 40 months. As shown in Table 2, significant differences in baseline characteristics were observed between survivors and deceased individuals. The mortality group had more participants aged > 80 years, females, singles, illiterates, and underweight individuals, but fewer current smokers and alcohol users. They also showed a higher prevalence of cardiovascular diseases. Significant intergroup differences were observed in serum albumin, lymphocyte count, platelet count, hemoglobin, C-reactive protein, blood urea nitrogen, total cholesterol, and triglycerides (all *P *< .001). All 6 nutrition-related indicators (HALP score, PNI, CPNI, TCBI, GNRI and BAR) also differed between 2 groups (all *P *< .001).

**Table 2:** The baseline characteristics of the Chinese older participants.

Characteristics	Total (n = 1691)	Death (n = 578)	Survey (n = 1113)	*P* value
Age (yrs), n (%)				< .001
≤ 80	603 (35.66)	57 (9.86)	546 (49.06)	
> 80	1088 (64.34)	521 (90.14)	567 (50.94)	
Sex, n (%)				< .001
Male	824 (48.73)	235 (40.66)	589 (52.92)	
Female	867 (51.27)	343 (59.34)	524 (47.08)	
Residence, n (%)				.967
Urban	333 (19.69)	113 (19.55)	220 (19.77)	
Rural	1358 (80.31)	465 (80.45)	893 (80.23)	
Marital status, n (%)				< .001
Married	682 (40.33)	127 (21.97)	555 (49.87)	
Single	1009 (59.67)	451 (78.03)	558 (50.13)	
Educational background, n (%)				< .001
Illiteracy	1046 (61.86)	440 (76.12)	606 (54.45)	
Literacy	645 (38.14)	138 (23.88)	507 (45.55)	
BMI (kg/m^2^), n (%)				< .001
underweight	315 (18.63)	159 (27.51)	156 (14.02)	
normal	958 (56.65)	312 (53.98)	646 (58.04)	
overweight	418 (24.72)	107 (18.51)	311 (27.94)	
Smoking status, n (%)				< .001
Never	1300 (76.88)	463 (80.10)	837 (75.20)	
Former	134 (7.92)	56 (9.69)	78 (7.01)	
Current	257 (15.20)	59 (10.21)	198 (17.79)	
Alcohol consumption, n (%)				.005
Never	1350 (79.83)	480 (83.04)	870 (78.17)	
Former	89 (5.26)	34 (5.88)	55 (4.94)	
Current	252 (14.90)	64 (11.07)	188 (16.89)	
Hypertension, n (%)				.270
Yes	1080 (63.87)	380 (65.74)	700 (62.89)	
No	611 (36.13)	198 (34.26)	413 (37.11)	
Diabetes, n (%)				.257
Yes	200 (11.83)	76 (13.15)	124 (11.14)	
No	1491 (88.17)	502 (86.85)	989 (88.86)	
Cardiovascular diseases, n (%)				.031
Yes	164 (9.70)	69 (11.94)	95 (8.54)	
No	1527 (90.30)	509 (88.06)	1018 (91.46)	
Stroke, n (%)				.768
Yes	105 (6.21)	34 (5.88)	71 (6.38)	
No	1586 (93.79)	544 (94.12)	1042 (93.62)	
C-reactive protein (mg/L)	1.16 (0.55, 2.52)	1.39 (0.62, 3.39)	1.04 (0.51, 2.17)	< .001
Plasma albumin (g/L)	43.10 (40.80, 45.05)	41.60 (39.00, 43.88)	43.80 (41.60, 45.50)	< .001
Lymphocyte count (10^9^/L)	1.82 (1.42, 2.37)	1.70 (1.30, 2.25)	1.90 (1.50, 2.40)	< .001
Platelet count (10^9^/L)	188.00 (149.00, 226.00)	184.00 (140.10, 224.75)	189.00 (153.00, 228.00)	.013
Hemoglobin (g/L)	129.00 (117.00, 141.00)	123.30 (112.00, 135.00)	132.00 (120.00, 144.00)	< .001
Blood urea nitrogen (mg/dL)	18.03 (14.77, 21.60)	19.14 (15.58, 22.74)	17.53 (14.39, 20.94)	< .001
Total cholesterol (mg/dL)	181.36 (158.55, 209.20)	177.30 (153.52, 203.79)	184.07 (162.41, 212.68)	< .001
Triglyceride (mg/dL)	95.66 (71.74, 132.85)	88.57 (69.97, 120.46)	99.20 (73.51, 138.17)	< .001
Nutrition-related indicators				
HALP score	56.36 (38.66, 76.40)	47.66 (33.99, 71.58)	59.74 (42.29, 78.71)	< .001
PNI	52.60 (49.20, 55.90)	50.83 (46.80, 54.00)	53.40 (50.35, 56.60)	< .001
CPNI	70.01 (64.88, 75.12)	72.17 (66.91, 77.74)	69.19 (64.07, 73.73)	< .001
TCBI	864.71 (579.48, 1352.87)	727.31 (494.87, 1080.42)	950.14 (641.16, 1479.81)	< .001
GNRI	102.90 (97.42, 107.07)	99.15 (93.74, 104.01)	104.52 (100.00, 108.23)	< .001
BAR	4.23 (3.45, 5.11)	4.67 (3.80, 5.73)	4.05 (3.29, 4.81)	< .001

Medians (interquartile ranges) was for continuous variables. Counts (percentages) was for categorical variables.

BAR = blood urea nitrogen to serum albumin ratio, BMI = body mass index, CPNI = cholesterol-modified prognostic nutritional index, GNRI = geriatric nutritional risk index, HALP = hemoglobin-albumin-lymphocyte-platelet, n = number of participants, PNI = prognostic nutritional index, TCBI = triglyceride-total cholesterol-body weight index.

### 3.2. Associations of nutrition-related indicators and all-cause mortality

**Figure 2.**
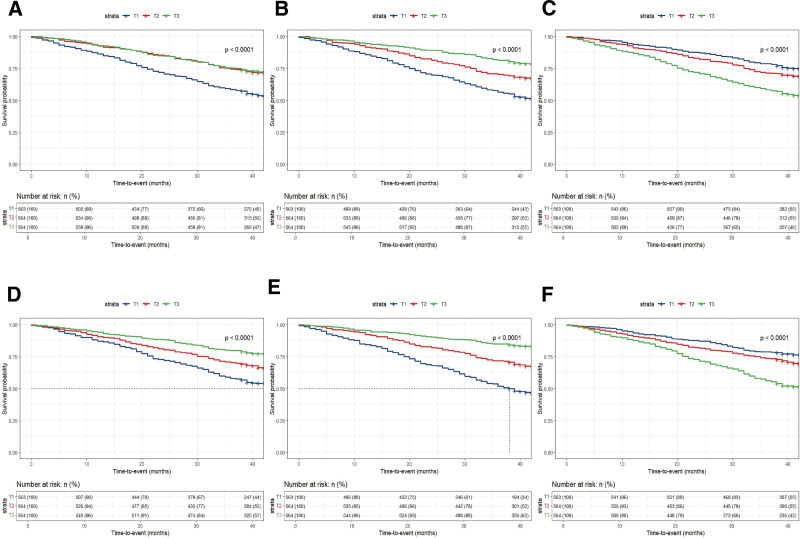
Kaplan–Meier survival curvess for elevated nutrition-related indicators: (A) HALP score; (B) PNI; (C) CPNI; (D) TCBI; (E) GNRI; and (F) BAR and all-cause mortality in Chinese elderly individuals. BAR = blood urea nitrogen to serum albumin ratio, CPNI = cholesterol-modified prognostic nutritional index/ cholesterol-modified PNI, GNRI = geriatric nutritional risk index, HALP = hemoglobin-albumin-lymphocyte-platelet, n = number of participants, PNI = prognostic nutritional index, TCBI = triglyceride-total cholesterol-body weight index.

Figure 2 presented Kaplan–Meier curves illustrating the associations between 6 nutrition-related indicators and all-cause mortality in older adults. Individuals in the lowest tertile (T1) of HALP score, PNI, TCBI, and GNRI exhibited significantly poorer overall survival (log-rank *P* < .001 for all). Conversely, those in the T1 of CPNI and BAR demonstrated significantly better overall survival (all log-rank *P* < .001).

Cox proportional hazards regression analyses further supported these findings (Table 3). In adjusted model 2, older adults in the highest tertile (T3) of HALP score (HR: 0.70, 95% CI: 0.57–0.85), PNI (HR: 0.53, 95% CI: 0.43–0.67), TCBI (HR: 0.63, 95% CI: 0.50–0.79), and GNRI (HR: 0.39, 95% CI: 0.30–0.52) had significantly lower risks of mortality compared to those in the T1. In contrast, individuals in the T3 groups for CPNI (HR: 1.63, 95% CI: 1.32–2.00) and BAR (HR: 1.78, 95% CI: 1.44–2.21) exhibited higher mortality risks relative to their T1 counterparts.

**Table 3:** Associations of nutrition-related indicators with the risk of all-cause mortality in Chinese older participants.

	Crude model HR (95% CI)	Adjusted model 1 HR (95% CI)	Adjusted model 2 HR (95% CI)
HALP score			
T1	1.00	1.00	1.00
T2	0.53 (0.44–0.65)	0.65 (0.53–0.79)	0.66 (0.54–0.81)
T3	0.52 (0.43–0.64)	0.70 (0.57–0.86)	0.70 (0.57–0.85)
*P* for trend	*P *< .001	*P *< .001	*P *< .001
Per standard deviation increase	0.79 (0.71–0.88)	0.90 (0.82–0.99)	0.90 (0.81–0.99)
PNI			
T1	1.00	1.00	1.00
T2	0.60 (0.50–0.72)	0.77 (0.63–0.93)	0.78 (0.64–0.94)
T3	0.37 (0.29–0.45)	0.52 (0.42–0.65)	0.53 (0.43–0.67)
*P* for trend	*P *< .001	*P *< .001	*P *< .001
Per standard deviation increase	0.60 (0.55–0.65)	0.71 (0.65–0.77)	0.71 (0.65–0.78)
CPNI			
T1	1.00	1.00	1.00
T2	1.28 (1.02–1.59)	1.24 (0.99–1.55)	1.23 (0.98–1.54)
T3	2.15 (1.75–2.64)	1.65 (1.34–2.03)	1.63 (1.32–2.00)
*P* for trend	*P *< .001	*P *< .001	*P *< .001
Per standard deviation increase	1.51 (1.39–1.65)	1.33 (1.22–1.45)	1.32 (1.21–1.44)
TCBI			
T1	1.00	1.00	1.00
T2	0.67 (0.56–0.81)	0.78 (0.65–0.95)	0.78 (0.64–0.95)
T3	0.42 (0.34–0.52)	0.64 (0.51–0.81)	0.63 (0.50–0.79)
*P* for trend	*P *< .001	*P *< .001	*P *< .001
Per standard deviation increase	0.67 (0.59–0.76)	0.84 (0.75–0.95)	0.83 (0.73–0.94)
GNRI			
T1	1.00	1.00	1.00
T2	0.52 (0.43–0.62)	0.64 (0.52–0.78)	0.65 (0.52–0.80)
T3	0.25 (0.20–0.31)	0.39 (0.30–0.52)	0.39 (0.30–0.52)
*P* for trend	*P *< .001	*P *< .001	*P *< .001
Per standard deviation increase	0.54 (0.50–0.59)	0.60 (0.54–0.67)	0.61 (0.54–0.67)
BAR			
T1	1.00	1.00	1.00
T2	1.34 (1.07–1.69)	1.18 (0.94–1.48)	1.19 (0.95–1.50)
T3	2.42 (1.97–2.98)	1.78 (1.44–2.20)	1.78 (1.44–2.21)
*P* for trend	*P *< .001	*P *< .001	*P *< .001
Per standard deviation increase	1.44 (1.35–1.54)	1.28 (1.19–1.37)	1.27 (1.18–1.36)

Crude model: did not adjust any covariates.

Adjusted model 1: adjusted for age, sex, residence, marital status, educational background, BMI, marital status, smoking status, and alcohol consumption.

Adjusted model 2: adjusted all covariates.

BAR = blood urea nitrogen to serum albumin ratio, BMI = body mass index, CI = confidence interval, CPNI = cholesterol-modified prognostic nutritional index, GNRI = geriatric nutritional risk index, HALP = hemoglobin-albumin-lymphocyte-platelet, HR = hazard ratio, PNI = prognostic nutritional index, TCBI = triglyceride-total cholesterol-body weight index.

### 3.3. A nonlinear dose-response relationship between nutrition-related indicators and all-cause mortality

**Figure 3.**
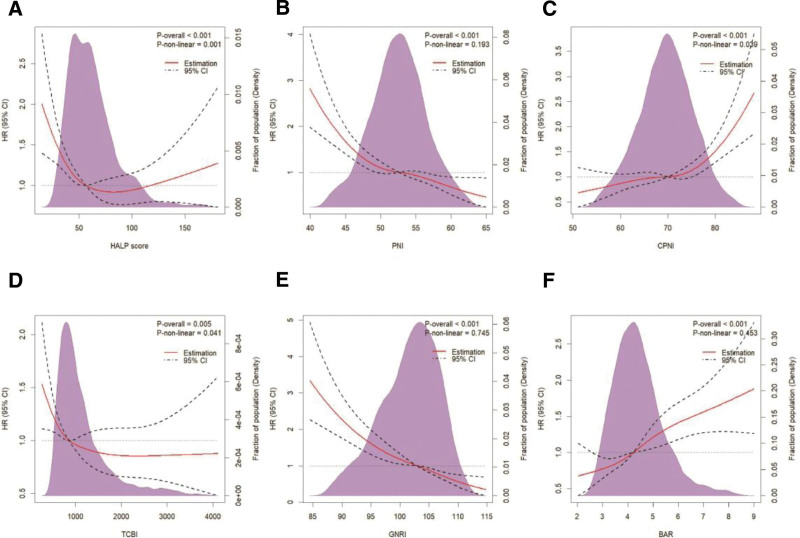
RCS regression with 4 knots was adjusted for age, sex, residence, marital status, educational background, BMI, smoking status, alcohol consumption, hypertension, diabetes, cardiovascular diseases, and stroke to examine the association between the nutrition-related indicators: (A) HALP score; (B) PNI; (C) CPNI; (D) TCBI; (E) GNRI; and (F) BAR and all-cause mortality in Chinese elderly individuals. BAR = blood urea nitrogen to serum albumin ratio, BMI = body mass index, CI = confidence interval, CPNI = cholesterol-modified prognostic nutritional index/ cholesterol-modified PNI, GNRI = geriatric nutritional risk index, HALP = hemoglobin-albumin-lymphocyte-platelet, HR = hazard ratio, PNI = prognostic nutritional index, RCS = restricted cubic spline, TCBI = triglyceride-total cholesterol-body weight index.

Nonlinear dose-response relationships between 6 nutrition-related indicators and all-cause mortality were analyzed using restricted cubic spline with 4 knots, with results visualized in Figure 3. Based on adjusted model 2, significant nonlinear associations were observed for HALP score, CPNI and TCBI (*P*_non−*linearity*_ < .05 for all), whereas PNI, GNRI, and BAR exhibited linear trends (*P*_non−*linearity*_ > .05 for all). Threshold effect analysis identified inflection points at 65.98 for HALP score, 74.34 for CPNI, and 1225.91 for TCBI (Tables S1, S2, and S3).

### 3.4. Comparison of nutritional-related indicators in predicting all-cause mortality

**Figure 4.**
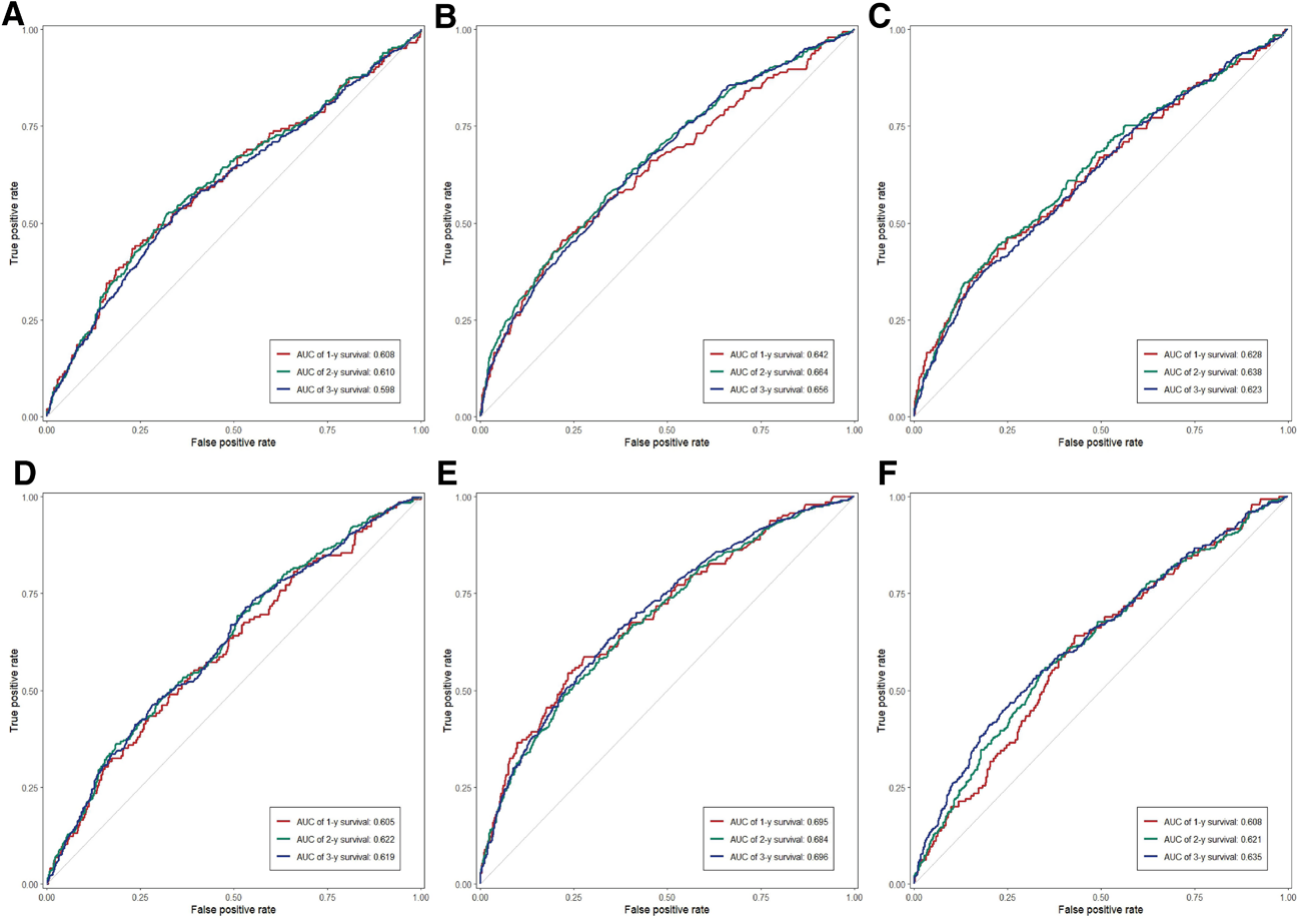
Predictive capability of time-dependent ROC assessment of nutrition-related indicators: (A) HALP score; (B) PNI; (C) CPNI; (D) TCBI; (E) GNRI; and (F) BAR for 1-, 2-, and 3-year all-cause mortality. AUC = area under the curve, BAR = blood urea nitrogen to serum albumin ratio, CPNI = cholesterol-modified prognostic nutritional index/ cholesterol-modified PNI, GNRI = geriatric nutritional risk index, HALP = hemoglobin-albumin-lymphocyte-platelet, PNI = prognostic nutritional index, ROC = receiver operating characteristic, TCBI = triglyceride-total cholesterol-body weight index.

Figure 4 presented time-dependent receiver operating characteristic curves comparing the predictive capacity of 6 nutrition-related indicators for all-cause mortality at 1-, 2-, and 3-year intervals. The results showed that GNRI exhibited the highest discriminative performance across all time points, with AUC values of 0.695, 0.684, and 0.696 at 1, 2, and 3 years, respectively. This was followed by PNI, which showed AUC values of 0.642, 0.664, and 0.656, respectively. Table S4 showed the Delong test results comparing AUC values among the indicators.

### 3.5. Subgroup and sensitivity analysis

Subgroup analyses stratified by age, sex, residence, BMI, hypertension, diabetes, cardiovascular diseases, and stroke were presented in Figures S1-S6. A higher HALP score was more strongly associated with reduced mortality risk among participants with diabetes (*P* for interaction = .04). In those with hypertension, higher PNI and lower CPNI showed a more substantial reduction in all-cause mortality risk. Additionally, higher GNRI was more strongly associated with reduced mortality among males and rural residents. No significant subgroup interactions were observed for TCBI or BAR (*P* for interaction > .05).

Sensitivity analyses excluding individuals aged over 100 years or those who died within 1 year of follow-up (Tables S5 and S6) revealed persistent inverse associations between HALP score, PNI, TCBI, and GNRI and all-cause mortality. In contrast, CPNI and BAR maintained persistent positive associations with all-cause mortality. These findings further confirm the robustness of the results derived from Cox regression analysis.

## 4. Discussion

In this prospective cohort study, we discussed the relationship between 6 nutrition-related indicators (HALP score, PNI, CPNI, TCBI, GNRI, and BAR) and the risk of all-cause mortality among community-dwelling older adults in China. Our key findings can be summarized as follows: HALP score, PNI, TCBI, and GNRI exhibited significant inverse associations with all-cause mortality, whereas CPNI and BAR showed significant positive associations; nonlinear dose-response relationships with mortality were observed for HALP score, CPNI and TCBI; among 6 nutrition-related indicators, GNRI demonstrated the strongest predictive performance for mortality at 1-, 2-, and 3-year intervals, followed by PNI.

Traditionally, dietitians recommend the Mini Nutritional Assessment to identify malnutrition in all geriatric settings.^[24]^ However, high clinician workloads and poor implementation, particularly in low- and middle-income country hospitals, have restricted their widespread use, resulting in unequal nutritional screening among older populations.^[12]^ This challenge has been of particular interest to our team in rehabilitation medicine, where long-term assessment of nutritional status in older adults is essential given its substantial influence on frailty, muscle performance, tissue repair, and functional recovery. To improve objectivity and feasibility, laboratory-based nutrition-related indicators have increasingly gained attention as alternative tools for assessing nutritional status and predicting clinical outcomes.

The HALP score, which incorporates hemoglobin, albumin, lymphocytes, and platelets, has established prognostic significance in geriatric patients with hip fractures, acute ischemic stroke, and osteoarthritis.^[15,25,26]^ Notably, earlier work,^[25]^ including our own, has identified a U-shaped association between the HALP score and mortality risk in older adults, likely attributable to the dual hazard of both low and high hemoglobin concentrations.^[27]^ The PNI, which integrates lymphocytes and albumin, serves as a reliable composite biomarker for nutritional status, inflammation, and immune function, and has been shown to predict both short- and long-term mortality across diverse geriatric populations.^[28–30]^ In contrast, the CPNI, a modified form of PNI incorporating cholesterol, was evaluated here for the first time in relation to mortality among older adults. Contrary to previous findings in oncological populations,^[16,31]^ the CPNI showed weaker predictive performance than the PNI in this study. Further analysis revealed significantly higher total cholesterol levels among survivors compared to non-survivors. This phenomenon is consistent with the well-recognized “cholesterol paradox” observed in geriatric populations,^[32,33]^ which may partly explain the limited prognostic performance of CPNI in this demographic. The TCBI exhibited an inverse association with mortality, consistent with recent studies in older patients with cardiovascular diseases, where it is increasingly recognized as a marker of frailty and poor prognosis.^[34]^ The BAR, a composite biomarker calculated as blood urea nitrogen divided by albumin, was positively associated with mortality, suggesting that an imbalance between protein catabolism and anabolism may contribute to poor prognosis in older adults.

The GNRI, proposed by Bouillanne in 2005, is an adaptation of the nutritional risk index specifically tailored for older adults.^[35]^ It integrates serum albumin concentration with anthropometric measures, including height, actual body weight, and ideal body weight, to assess nutritional status.^[18]^ In geriatric populations, the GNRI has been widely applied in the management of chronic diseases such as stroke, diabetes, and heart failure,^[36–38]^ where lower values are consistently linked to higher mortality, frequent hospitalizations, and functional decline. Our study highlights GNRI as the most effective predictor of 1-, 2-, and 3-year mortality among community-dwelling older adults. Its predictive strength lies in its ability to capture both protein-energy malnutrition and deviations from normal BMI by reflecting protein reserves and underweight relative to ideal body weight, thereby providing a more accurate and comprehensive assessment of current nutritional status.

In older adults, malnutrition primarily stems from inadequate food intake caused by age-related physiological changes, including taste bud atrophy, olfactory degeneration, and oral health problems.^[39,40]^ These impairments diminish the enjoyment of food and limit the consumption of nutrient-dense foods requiring chewing, such as meat, fruits, and vegetables.

In addition, malnutrition is strongly associated with chronic low-grade inflammatory states, a hallmark of aging. Chronic low-grade inflammatory states is characterized by elevated circulating levels of pro-inflammatory cytokines, including interleukin-6, tumor necrosis factor-α, and C-reactive protein.^[41]^ These cytokines collectively contribute to malnutrition through multiple mechanisms: suppressing orexigenic neuronal pathways while stimulating anorexigenic pathways to inhibit hunger signals^[42,43]^; disrupting protein homeostasis via modulation of the ubiquitin-proteasome system, the autophagy-lysosomal pathway, and the mammalian target of rapamycin-dependent protein synthesis pathways^[44–46]^; and disrupting gut microbiota equilibrium and increasing intestinal permeability, which impair micronutrient absorption.^[47]^

Adequate protein and micronutrient intake are critical for the development, maintenance, and optimal function of immune cells.^[48]^ Proteins provide the structural foundation for immune cells, antibodies, and signaling molecules. Thus, protein-energy malnutrition weakens both cell-mediated and humoral immune responses, compromising host defenses and inflammatory regulation. Micronutrients such as zinc, selenium, and vitamins C and E play indispensable roles in maintaining oxidative homeostasis by scavenging reactive oxygen species.^[49]^ Deficiencies impair antioxidant defenses, leading to reactive oxygen species accumulation, which activates Nuclear factor-kappa B signaling and upregulate pro-inflammatory cytokines.^[49]^ This process perpetuates and amplifies preexisting chronic low-grade inflammation, ultimately contributing to age-related diseases. Moreover, micronutrients are essential for immune cell proliferation, differentiation, and functional specialization. For example, zinc maintains thymic integrity and T- and B-lymphocyte numbers; vitamin C enhances neutrophil chemotaxis, phagocytosis, and the clearance of apoptotic cells; and vitamin D modulates both innate and adaptive responses through its receptor on monocytes and T-cells.^[48,50,51]^ Altogether, malnutrition accelerates immunosenescence and inflammaging, which in turn increases the risks of morbidity and mortality in older adults.

While this study is the first to compare 6 novel nutrition-related indicators for assessing adverse outcomes among community-dwelling older adults in China, several limitations should be acknowledged. First, the nutritional-related indicators were derived from a single baseline blood sample, which does not capture longitudinal changes in nutritional status that may provide important prognostic information. Second, despite rigorous multivariable adjustment, residual confounding from unmeasured variables may remain. Finally, as the study population was drawn from Chinese communities, caution is warranted when generalizing these findings to other populations.

## 5. Conclusion

In conclusion, this prospective cohort study of Chinese adults aged 65 and older identified significant associations between nutrition-related indicators and all-cause mortality. Specifically, lower HALP score, PNI, TCBI, and GNRI, as well as higher CPNI and BAR, were linked to increased risk of all-cause mortality. Importantly, GNRI exhibited the strongest predictive performance among the 6 indicators. These findings underscore the pivotal role of nutritional status in shaping health outcomes in older populations. Given its cost-effectiveness and prognostic utility, integrating GNRI into routine geriatric assessments, particularly in resource-limited settings, may enable earlier identification of high-risk individuals and support targeted nutritional interventions to improve survival outcomes.

## Author contributions

**Data curation:** Tian Hu, Yuhan Ye, Zelong Zhao.

**Methodology:** Tian Hu.

**Project administration:** Zheng Hu.

**Supervision:** Zheng Hu.

**Visualization:** Tian Hu, Yuhan Ye.

**Writing – original draft:** Tian Hu, Yuhan Ye, Zelong Zhao.

**Writing – review & editing:** Zheng Hu.
























