# Supplementary material for: Six novel nutritional-related indicators predict 3-year all-cause mortality among community-dwelling older adults in China: A cohort study based on CLHLS from 2014 to 2018
Source: Medicine (Baltimore). 2026 May 22;105(21):e48952. doi: 10.1097/MD.0000000000048952 (PMC13200928; doi:10.1097/MD.0000000000048952)
Supplement: Supplementary file 1 [file medi-105-e48952-s001.docx]

**Table S1. Threshold effect analysis between HALP score and all-cause mortality.**

| **All-cause mortality** | **HALP score** | |
| --- | --- | --- |
|  | HR (95%CI) | *P* value |
| Model 1: Fitting model of standard multi-factor Cox regression analysis model | 0.995(0.992-0.999) | 0.004 |
| Model 2: Fitting model of two-piecewise multi-factor Cox regression analysis model |  |  |
| Inflection point | 65.98 |  |
| < 65.98 | 0.985(0.979-0.991) | < 0.001 |
| > 65.98 | 1.004(0.999-1.008) | 0.129 |
| *P* for likelihood ratio test | < 0.001 | |

CI = confidence interval, HALP = hemoglobin-albumin-lymphocyte-platelet, HR = hazard ratio.
