# Supplementary material for: Six novel nutritional-related indicators predict 3-year all-cause mortality among community-dwelling older adults in China: A cohort study based on CLHLS from 2014 to 2018
Source: Medicine (Baltimore). 2026 May 22;105(21):e48952. doi: 10.1097/MD.0000000000048952 (PMC13200928; doi:10.1097/MD.0000000000048952)
Supplement: Supplementary file 2 [file medi-105-e48952-s002.docx]

**Table S4. DeLong test for time-dependent ROC curve comparisons of different nutrition-related indicators.**

| Nutrition-related indicators | DeLong test adjusted *P*-value | | |
| --- | --- | --- | --- |
|  | AUC of 1-y survival | AUC of 2-y survival | AUC of 3-y survival |
| HALP score vs. PNI | 0.239 | < 0.001 | < 0.001 |
| HALP score vs. CPNI | 0.629 | 0.187 | 0.149 |
| HALP score vs. TCBI | 1.000 | 0.891 | 0.526 |
| HALP score vs. GNRI | 0.005 | < 0.001 | < 0.001 |
| HALP score vs. BAR | 1.000 | 0.926 | 0.154 |
| PNI vs. CPNI | 0.559 | 0.022 | < 0.001 |
| PNI vs. TCBI | 0.421 | 0.091 | 0.075 |
| PNI vs. GNRI | 0.042 | 0.358 | 0.007 |
| PNI vs. BAR | 0.500 | 0.111 | 0.495 |
| CPNI vs. TCBI | 0.822 | 0.831 | 0.993 |
| CPNI vs. GNRI | 0.023 | 0.025 | < 0.001 |
| CPNI vs. BAR | 0.844 | 0.783 | 0.850 |
| TCBI vs. GNRI | < 0.001 | 0.001 | < 0.001 |
| TCBI vs. BAR | 0.999 | 1.000 | 0.725 |
| GNRI vs. BAR | 0.005 | 0.006 | 0.002 |

AUC = area under the curve, BAR = blood urea nitrogen to serum albumin ratio, CPNI = cholesterol-modified prognostic nutritional index, GNRI = geriatric nutritional risk index, HALP = hemoglobin-albumin-lymphocyte-platelet, PNI = prognostic nutritional index, ROC = receiver operating characteristic, TCBI = triglyceride-total cholesterol-body weight index.
