# Supplementary material for: Six novel nutritional-related indicators predict 3-year all-cause mortality among community-dwelling older adults in China: A cohort study based on CLHLS from 2014 to 2018
Source: Medicine (Baltimore). 2026 May 22;105(21):e48952. doi: 10.1097/MD.0000000000048952 (PMC13200928; doi:10.1097/MD.0000000000048952)
Supplement: Supplementary file 3 [file medi-105-e48952-s003.docx]

**
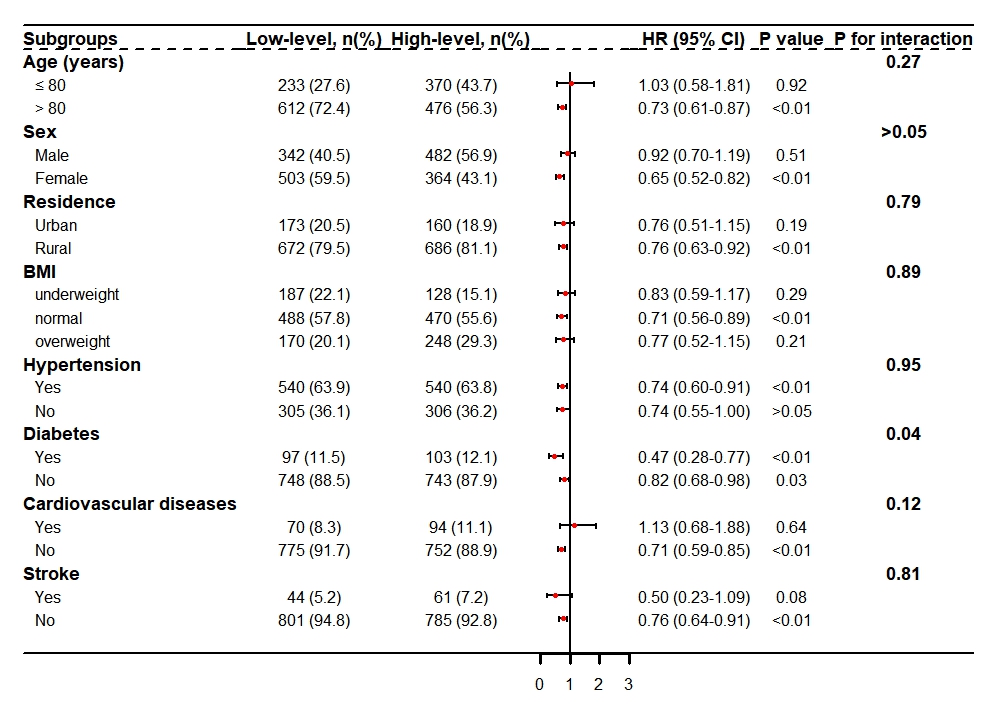
Fig S1. The relationship between HALP score and all-cause mortality, stratified by age, sex, residence, BMI, hypertension, diabetes, cardiovascular diseases and stroke.** BMI = body mass index, CI = confidence interval, HALP = hemoglobin-albumin-lymphocyte-platelet, HR = hazard ratio.
