# Supplementary material for: Six novel nutritional-related indicators predict 3-year all-cause mortality among community-dwelling older adults in China: A cohort study based on CLHLS from 2014 to 2018
Source: Medicine (Baltimore). 2026 May 22;105(21):e48952. doi: 10.1097/MD.0000000000048952 (PMC13200928; doi:10.1097/MD.0000000000048952)
Supplement: Supplementary file 4 [file medi-105-e48952-s004.docx]

**Table S5.** **Sensitivity analysis: exclusion of individuals aged over 100 years.**

|  | Crude model HR (95% CI) | Adjusted model 1 HR (95% CI) | Adjusted model 2 HR (95% CI) |
| --- | --- | --- | --- |
| **HALP score** |  |  |  |
| T1 | 1.00 | 1.00 | 1.00 |
| T2 | 0.53 (0.42-0.66) | 0.65 (0.51-0.81) | 0.66 (0.53-0.84) |
| T3 | 0.58 (0.47-0.73) | 0.76 (0.61-0.96) | 0.76 (0.61-0.95) |
| *P* for trend | *P* < 0.001 | *P* = 0.012 | *P* = 0.013 |
| Per standard deviation increase | 0.82 (0.73-0.93) | 0.93 (0.83-1.03) | 0.92 (0.82-1.03) |
| **PNI** |  |  |  |
| T1 | 1.00 | 1.00 | 1.00 |
| T2 | 0.62 (0.51-0.77) | 0.84 (0.67-1.04) | 0.86 (0.69-1.07) |
| T3 | 0.38 (0.30-0.48) | 0.54 (0.42-0.68) | 0.55 (0.43-0.71) |
| *P* for trend | *P* < 0.001 | *P* < 0.001 | *P* < 0.001 |
| Per standard deviation increase | 0.62 (0.52-0.68) | 0.73 (0.66-0.80) | 0.73 (0.67-0.81) |
| **CPNI** |  |  |  |
| T1 | 1.00 | 1.00 | 1.00 |
| T2 | 1.19 (0.93-1.52) | 1.16 (0.90-1.48) | 1.15 (0.90-1.47) |
| T3 | 1.99 (1.59-2.51) | 1.55 (1.23-1.95) | 1.51 (1.20-1.91) |
| *P* for trend | *P* < 0.001 | *P* < 0.001 | *P* < 0.001 |
| Per standard deviation increase | 1.46 (1.32-1.61) | 1.29 (1.17-1.42) | 1.28 (1.16-1.41) |
| **TCBI** |  |  |  |
| T1 | 1.00 | 1.00 | 1.00 |
| T2 | 0.67 (0.54-0.83) | 0.77 (0.62-0.96) | 0.76 (0.61-0.95) |
| T3 | 0.46 (0.36-0.58) | 0.68 (0.53-0.88) | 0.66 (0.51-0.86) |
| *P* for trend | *P* < 0.001 | *P* = 0.002 | *P* = 0.001 |
| Per standard deviation increase | 0.71 (0.62-0.80) | 0.87 (0.76-0.99) | 0.85 (0.74-0.97) |
| **GNRI** |  |  |  |
| T1 | 1.00 | 1.00 | 1.00 |
| T2 | 0.52 (0.42-0.65) | 0.65 (0.51-0.83) | 0.67 (0.52-0.85) |
| T3 | 0.27 (0.21-0.35) | 0.41 (0.30-0.55) | 0.42 (0.31-0.56) |
| *P* for trend | *P* < 0.001 | *P* < 0.001 | *P* < 0.001 |
| Per standard deviation increase | 0.55 (0.51-0.61) | 0.61 (0.54-0.68) | 0.61 (0.54-0.69) |
| **BAR** |  |  |  |
| T1 | 1.00 | 1.00 | 1.00 |
| T2 | 1.35 (1.05-1.74) | 1.19 (0.93-1.54) | 1.21 (0.94-1.56) |
| T3 | 2.41 (1.91-3.04) | 1.80 (1.42-2.29) | 1.79 (1.41-2.28) |
| *P* for trend | *P* < 0.001 | *P* < 0.001 | *P* < 0.001 |
| Per standard deviation increase | 1.43 (1.33-1.54) | 1.27 (1.17-1.37) | 1.26 (1.16-1.36) |

Crude model: did not adjust any covariates.

Adjusted model 1: adjusted for age, sex, residence, marital status, educational background, BMI, marital status, smoking status, and alcohol consumption.

Adjusted model 2: adjusted all covariates.

BAR = blood urea nitrogen to serum albumin ratio, BMI = body mass index, CI = confidence interval, CPNI = cholesterol-modified prognostic nutritional index, GNRI = geriatric nutritional risk index, HALP = hemoglobin-albumin-lymphocyte-platelet, HR = hazard ratio, PNI = prognostic nutritional index, TCBI = triglyceride-total cholesterol-body weight index.
