# Supplementary material for: Six novel nutritional-related indicators predict 3-year all-cause mortality among community-dwelling older adults in China: A cohort study based on CLHLS from 2014 to 2018
Source: Medicine (Baltimore). 2026 May 22;105(21):e48952. doi: 10.1097/MD.0000000000048952 (PMC13200928; doi:10.1097/MD.0000000000048952)
Supplement: Supplementary file 10 [file medi-105-e48952-s010.docx]

**Table S2. Threshold effect analysis between CPNI levels and all-cause mortality.**

| **All-cause mortality** | **CPNI** | |
| --- | --- | --- |
|  | HR (95%CI) | *P* value |
| Model 1: Fitting model of standard multi-factor Cox regression analysis model | 1.033(1.021-1.045) | < 0.001 |
| Model 2: Fitting model of two-piecewise multi-factor Cox regression analysis model |  |  |
| Inflection point | 74.34 |  |
| < 74.34 | 1.014(0.997-1.031) | 0.118 |
| > 74.34 | 1.069(1.041-1.098) | < 0.001 |
| *P* for likelihood ratio test | 0.007 | |

CI = confidence interval, CPNI = cholesterol-modified prognostic nutritional index, HR = hazard ratio.
