# Supplementary material for: Six novel nutritional-related indicators predict 3-year all-cause mortality among community-dwelling older adults in China: A cohort study based on CLHLS from 2014 to 2018
Source: Medicine (Baltimore). 2026 May 22;105(21):e48952. doi: 10.1097/MD.0000000000048952 (PMC13200928; doi:10.1097/MD.0000000000048952)
Supplement: Supplementary file 11 [file medi-105-e48952-s011.docx]

**Table S3. Threshold effect analysis between TCBI levels and all-cause mortality.**

| **All-cause mortality** | **TCBI** | |
| --- | --- | --- |
|  | HR (95%CI) | *P* value |
| Model 1: Fitting model of standard multi-factor Cox regression analysis model | 0.999(0.999-1.000) | 0.014 |
| Model 2: Fitting model of two-piecewise multi-factor Cox regression analysis model |  |  |
| Inflection point | 1225.91 |  |
| < 1225.91 | 0.999(0.999-1.000) | 0.001 |
| > 1225.91 | 1.000(1.000-1.000) | 0.793 |
| *P* for likelihood ratio test | 0.009 | |

CI = confidence interval, HR = hazard ratio, TCBI = triglyceride-total cholesterol-body weight index.
