# Supplementary material for: Six novel nutritional-related indicators predict 3-year all-cause mortality among community-dwelling older adults in China: A cohort study based on CLHLS from 2014 to 2018
Source: Medicine (Baltimore). 2026 May 22;105(21):e48952. doi: 10.1097/MD.0000000000048952 (PMC13200928; doi:10.1097/MD.0000000000048952)
Supplement: Supplementary file 12 [file medi-105-e48952-s012.docx]

**Table S6. Sensitivity analysis: exclusion of individuals who died within one year of follow-up.**

|  | Crude model HR (95% CI) | Adjusted model 1 HR (95% CI) | Adjusted model 2 HR (95% CI) |
| --- | --- | --- | --- |
| **HALP score** |  |  |  |
| T1 | 1.00 | 1.00 | 1.00 |
| T2 | 0.54 (0.43-0.68) | 0.65 (0.52-0.82) | 0.66 (0.52-0.84) |
| T3 | 0.54 (0.43-0.68) | 0.72 (0.57-0.91) | 0.71 (0.56-0.90) |
| *P* for trend | *P* < 0.001 | *P* = 0.003 | *P* = 0.003 |
| Per standard deviation increase | 0.78 (0.69-0.88) | 0.88 (0.79-0.99) | 0.88 (0.79-0.98) |
| **PNI** |  |  |  |
| T1 | 1.00 | 1.00 | 1.00 |
| T2 | 0.61 (0.49-0.76) | 0.77 (0.62-0.97) | 0.78 (0.62-0.98) |
| T3 | 0.36 (0.28-0.47) | 0.50 (0.39-0.65) | 0.51 (0.39-0.66) |
| *P* for trend | *P* < 0.001 | *P* < 0.001 | *P* < 0.001 |
| Per standard deviation increase | 0.62 (0.56-0.68) | 0.71 (0.65-0.79) | 0.71 (0.65-0.79) |
| **CPNI** |  |  |  |
| T1 | 1.00 | 1.00 | 1.00 |
| T2 | 1.22 (0.94-1.58) | 1.19 (0.92-1.54) | 1.19 (0.91-1.54) |
| T3 | 2.12 (1.67-2.69) | 1.65 (1.29-2.09) | 1.63 (1.28-2.07) |
| *P* for trend | *P* < 0.001 | *P* < 0.001 | *P* < 0.001 |
| Per standard deviation increase | 1.46 (1.32-1.61) | 1.29 (1.16-1.42) | 1.28 (1.16-1.42) |
| **TCBI** |  |  |  |
| T1 | 1.00 | 1.00 | 1.00 |
| T2 | 0.66 (0.53-0.82) | 0.74 (0.59-0.93) | 0.74 (0.59-0.93) |
| T3 | 0.41 (0.32-0.53) | 0.58 (0.45-0.77) | 0.57 (0.43-0.75) |
| *P* for trend | *P* < 0.001 | *P* < 0.001 | *P* < 0.001 |
| Per standard deviation increase | 0.66 (0.57-0.76) | 0.80 (0.69-0.93) | 0.79 (0.68-0.91) |
| **GNRI** |  |  |  |
| T1 | 1.00 | 1.00 | 1.00 |
| T2 | 0.53 (0.43-0.66) | 0.62 (0.49-0.80) | 0.63 (0.49-0.81) |
| T3 | 0.24 (0.18-0.31) | 0.34 (0.25-0.47) | 0.34 (0.25-0.47) |
| *P* for trend | *P* < 0.001 | *P* < 0.001 | *P* < 0.001 |
| Per standard deviation increase | 0.57 (0.52-0.62) | 0.62 (0.55-0.69) | 0.61 (0.54-0.69) |
| **BAR** |  |  |  |
| T1 | 1.00 | 1.00 | 1.00 |
| T2 | 1.31 (1.01-1.71) | 1.15 (0.88-1.51) | 1.16 (0.88-1.52) |
| T3 | 2.61 (2.04-3.32) | 1.98 (1.54-2.53) | 1.97 (1.54-2.53) |
| *P* for trend | *P* < 0.001 | *P* < 0.001 | *P* < 0.001 |
| Per standard deviation increase | 1.47 (1.36-1.58) | 1.31 (1.21-1.42) | 1.30 (1.20-1.41) |

Crude model: did not adjust any covariates.

Adjusted model 1: adjusted for age, sex, residence, marital status, educational background, BMI, marital status, smoking status, and alcohol consumption.

Adjusted model 2: adjusted all covariates.

BAR = blood urea nitrogen to serum albumin ratio, BMI = body mass index, CI = confidence interval, CPNI = cholesterol-modified prognostic nutritional index, GNRI = geriatric nutritional risk index, HALP = hemoglobin-albumin-lymphocyte-platelet, HR = hazard ratio, PNI = prognostic nutritional index, TCBI = triglyceride-total cholesterol-body weight index.
